# Provider Attitudes and Perceptions on Using Artificial Intelligence in Colonoscopy: A Systematic Review and Meta-Analysis

**DOI:** 10.1016/j.gastha.2025.100746

**Published:** 2025-07-11

**Authors:** Saeed Soleymanjahi, Niroop Rajashekar, Sunny Chung, Alyssa A. Grimshaw, Mary Jo K. Tvedt, Farid Foroutan, Shahnaz Sultan, Dennis L. Shung, Jennifer M. Kolb

**Affiliations:** 1 Division of Gastroenterology, Mass General Brigham, Harvard School of Medicine, Boston, Massachusetts; 2 Department of Medicine, Yale School of Medicine, New Haven, Connecticut; 3 Section of Digestive Diseases, Department of Medicine, Yale School of Medicine, New Haven, Connecticut; 4 Cushing/Whitney Medical Library, Yale University, New Haven, Connecticut; 5 School of Science, Suffolk University & Mass General Brigham, Harvard School of Medicine, Boston, Massachusetts; 6 Ted Rogers Centre for Heart Research, University Health Network, Toronto, Canada; 7 Division of Gastroenterology, Hepatology and Nutrition, University of Minnesota, Minneapolis, Minnesota; 8 Section of Digestive Diseases, Clinical and Translational Research Accelerator, Department of Biomedical Informatics and Data Science, Department of Medicine, Yale School of Medicine, New Haven, Connecticut; 9 Vatche and Tamar Manoukian Division of Digestive Diseases, David Geffen School of Medicine at UCLA, Los Angeles, California; 10 Division of Gastroenterology, Hepatology and Parenteral Nutrition, Greater Los Angeles VA Healthcare System

**Keywords:** Artificial intelligence, Colonoscopy, Adenoma detection rate

## Abstract

**Background and Aims:**

Colonoscopy is the gold standard screening modality for colorectal cancer; however, it is operator-dependent and reliant on exam quality. Incorporating artificial intelligence (AI) into colonoscopy may improve adenoma detection and clinical outcomes, but this is a sociotechnical challenge that requires effective human–AI teaming incorporating provider attitudes.

**Methods:**

We conducted a systematic review of studies evaluating attitudes and perspectives of providers toward AI-assisted colonoscopy. Participant responses to outcome questions of interest were combined across the studies to calculate pooled proportion (Pp) and 95% confidence interval (CI). Top-ranked perceived advantages and disadvantages in each study were defined as the items that >50% of the study participants voted for.

**Results:**

Out of 2044 abstracts screened, 13 studies were included representing 1538 providers who were mostly gastroenterologists or trainees and 25%–100% had direct experience using AI in a clinical setting. Overall, a large majority were interested in using AI (Pp = 80%, 95% CI 70%–89%, n = 8 studies) and believed it can improve adenoma or polyp detection rate (Pp = 74%, 95% CI 68%–80%, n = 4 studies). Among 5 studies addressing financial implications, about half were concerned about the cost of using AI (52%, 95% CI 24%–79%). An average of 38% of respondents (95% CI 9%–73%) from 4 studies raised concern regarding accountability for misdiagnosis. High number of false positives and an absence of clinical guidelines were top-ranked perceived disadvantages in 2 studies.

**Conclusion:**

Most gastroenterology providers expressed interest in using AI systems with colonoscopy and believed it can improve adenoma detection rate. Cost, high number of false positives, and lack of professional society guidelines were among top perceived concerns.

## Introduction

Colorectal cancer (CRC) is the fourth most common cancer and second leading cause of cancer-related mortality.^1^^,^^2^ CRC screening programs have resulted in reduction of cancer incidence and mortality through detection and removal of precancerous lesions.^3^ The success of these procedures depends on colonoscopy quality with the most widely used quality indicator being the adenoma detection rate (ADR).^4^^,^^5^ Higher ADRs have been tied to fewer postcolonoscopy CRCs and lower mortality, though there still remains a miss rate of 26%.^6^^,^^7^ Therefore, strategies to augment the effectiveness of colonoscopy have largely focused on increasing ADR through mucosal exposure devices and techniques to improve visualization, improved bowel preparation, and spending adequate time inspecting.

Artificial intelligence (AI) and machine learning–based approaches have demonstrated a key role in colonoscopy by improving visual inspection to mitigate operator dependency and reduce adenoma miss rate.^8^ More advanced AI systems based on convolutional neural networks have been developed and interrogated in colonoscopy to identify polyps in real time, called computer-aided detection (CADe).^9^ Several randomized clinical trials have demonstrated clinical effectiveness of different commercially available CADe platforms by comparing them with conventional colonoscopy.^10^, ^11^, ^12^, ^13^ A recent meta-analysis including 44 randomized clinical trials demonstrated improved adenoma and polyp detection and decreased adenoma miss rate using CADe that was more pronounced in providers with low (<25%) baseline ADR.^14^ Performance of CADe on detecting clinically more relevant lesions requires further investigation.^15^ In contrast to these controlled research environments, data from real-world nonrandomized studies indicate minimal added benefit with CADe.^16^

Another application of AI in colonoscopy is computer-aided diagnosis (CADx), which is developed to help predict polyp histology during colonoscopy and prevent unnecessary removal of nonneoplastic polyps. Although there are currently no adequately designed or powered randomized controlled trials to evaluate CADx, available studies do not suggest a clear benefit for colon polyps using the resect and discard strategy.^17^^,^^18^

Whether CADe and CADx should be widely implemented in colonoscopy depends on more than just improvement in outcomes and reduction of harms. The integration of AI systems into clinical care represents a sociotechnical challenge of human–AI teaming that requires an understanding of how humans perceive the technology.^19^ Acceptance of these new technologies is contingent on the willingness and attitude of providers in the endoscopy community. Interest and confidence in these systems is critical for building trust in the technology to ensure effective use in clinical care. Obtaining insights into various aspects of providers’ perspective on AI-assisted colonoscopy can aid expert societies in providing recommendations customized to address the specific needs and major concerns of the clinicians. To address this gap, we conducted a systematic review to summarize gastroenterology providers’ perspectives toward integrating AI-assisted colonoscopy into their practice related to interest, willingness, and perceived concerns and barriers.

## Methods

### Registration of Review Protocol

The Preferred Reporting Items for Systematic Reviews and Meta-analyses statement for reporting was used for this study (Table A1).^20^ The protocol was registered a priori (CRD42023466796). Institutional review board approval was not required for this study, as it is a systematic review and meta-analysis of previously published data. No new data involving human participants were collected or analyzed.

### Search Strategies and Study Selection

An exhaustive search of the literature was conducted in Cochrane Library, Google Scholar, Ovid Embase, Ovid MEDLINE, PubMed, Scopus, and Web of Science Core Collection databases to find relevant articles published from the inception of each database to July 26, 2024. Databases were searched using a combination of keywords and controlled vocabulary for AI, colonoscopy, and trust or acceptance. The search was not limited by language, publication type, or year (see Table A2 for full search strategy). The search was peer-reviewed by a second medical librarian using the Peer Review of Electronic Search Strategies.^21^ Forward and backward citation chasing was performed using CitationChaser, ResearchRabbit, and SpiderCite to identify additional relevant studies not retrieved by the database search.^22^

Search results from all databases were imported into an Endnote 20 library. Duplicates were removed using the Yale Reference Deduplicator. The deduplicated results were then imported into Covidence, a systematic review software for screening.

### Study Selection and Inclusion and Exclusion Criteria

Two independent screeners (S.S., J.M.K.) performed title/abstract review followed by full text review. Screening disagreements were resolved by a third investigator (D.L.S.). We included studies that reported any providers’ perspective toward adopting AI-assisted colonoscopy (either CADe or CADx). For conference abstracts, we reached out to the corresponding author of the abstract to see if they have concluded the project and whether we can use their final data in our review. If not available, abstracts were excluded from the review.

### Data Extraction

Two independent reviewers (S.S., J.M.K.) performed the data extraction. Any conflict was discussed between the 2 reviewers and if unresolved, addressed by the third reviewer (D.L.S.). Study characteristics included the year of publication, country of origin, time period of data collection, survey method, number of participants and response rate (%), clinical setting (eg private practice, academic center), exclusion criteria, and characteristics of study participants in terms of specialty (eg gastroenterology [GI], surgery), title (eg physician, nurse endoscopist), level of experience (eg senior attending, fellow), experience of using AI in the clinical setting, and level of familiarity with AI.

Outcome measures included providers’ (1) interest, satisfaction, or willingness to use AI in colonoscopy, (2) perceived advantages to using AI, and (3) disadvantages, barriers, and concerns toward using AI in their clinical practice. The primary approach was to report the proportion of study participants with a positive response to survey questions related to each outcome measure. When these data were not available, narrative descriptions were provided. We also present comparison data among subgroups of participants categorized based on level of experience (senior provider vs fellow), clinical setting (academic vs private practice), and familiarity with or experience of using AI. We extracted the top ranked perceived advantages and disadvantages in each study. Top rank was defined as an advantage or disadvantage item that >50% of the study responders voted for.

### Risk of Bias Assessment

Two reviewers (S.S., J.M.K.) independently evaluated risk of bias using Joanna Briggs Institute (JBI) critical appraisal checklist and any discrepancies were addressed by discussion. The JBI checklist consists of 10 items related to philosophy of the study and study question, method, presentation, and interpretation of data, declaring conflict of interest, representation of the study participants, ethical approval, and conclusion supported by the study findings.^23^

### Data Synthesis

When data were available from more than 2 studies, we used random-effect meta-analysis to combine and synthesize the pooled proportion (Pp) with 95% confidence interval (CI). The heterogeneity among studies for the outcome measures were assessed based on visual inspection of the forest plots and using the *I*^2^ statistics that explains the proportion of the variation in data due to among-study differences. Cut-off points of 25% and 75% were considered to define moderate and high heterogeneity, respectively. When high heterogeneity was noted among studies, we compared the studies in terms of characteristics of interest to find those that might be related to the heterogeneity in the outcome measure. Given the low number of studies included in each data synthesis, we did not conduct statistical tests to evaluate publication bias.

In addition to the proportion data, we documented the top-ranked perceived advantages and disadvantage of using AI in each study, defined as when >50% of the study participants voted for that item. We grouped items together if they fit a similar concept (eg, shorter endoscopy time and time saving for the physicians) and counted the total number of studies that ranked any of the related items among top advantages or disadvantages.

## Results

### Search Strategy

Of the initial 2044 records screened, 41 were selected for full text retrieval. After removing abstracts, review studies and studies with wrong design, intervention, outcome, or patient population, or studies with duplicate data (Table A3), we included 11 studies that met our inclusion criteria. Two additional papers were found through reference chasing for a total of 13 included studies (Figure A1).^24^, ^25^, ^26^, ^27^, ^28^, ^29^, ^30^, ^31^, ^32^, ^33^, ^34^, ^35^, ^36^

### Study Characteristics

All 13 included articles were survey studies (12 conducted online and 1 in-person using paper questionnaire^34^) conducted between 2018 and 2023 (Table) and including 1538 participants in total. Four studies were conducted in the US,^26^^,^^28^^,^^35^^,^^36^ 2 in the UK,^25^^,^^27^ 4 in Asia-Pacific region,^24^^,^^30^, ^31^, ^32^ 2 in the Netherlands,^33^^,^^34^ and 1 included respondents from a global audience.^29^ The number of participants per study ranged from 16 to 374 and the response rate ranged from 3.7% to 92%. In most of the studies, the participants were senior gastroenterologists and gastroenterology fellows. Two studies from the UK included endoscopists with a nursing background^25^^,^^27^ and 2 studies included senior surgeons and surgical trainees.^24^^,^^27^ The respondents were from a wide range of clinical settings including academic setting, tertiary and secondary care centers, and private practice.^26^^,^^35^ Specific age, sex, years of experience, and ADR (when available) is summarized in Table A4. In 7 studies, a proportion of the participants (25%–100%) had direct experience with using AI in a clinical setting.^24^^,^^29^^,^^30^^,^^32^, ^33^, ^34^^,^^36^ In 4 studies, varying proportion (0%–100%) of the participants reported they were familiar with AI or had prior formal training by attending an AI conference or course, but did not have experience with using it in a clinical setting.^25^, ^26^, ^27^, ^28^ The studies that included more senior providers tended to have greater familiarity/experience with AI among their participants compared to other studies (*rho* 0.77, *P* = .021, n = 9 studies). Four studies evaluated respondents view toward a specific AI platform (eg GI Genius).^28^^,^^31^^,^^32^^,^^36^

**Table:** Study Characteristics

Author, year	Country	Study period	Survey method	Participants N (*response rate %*)	Study population	Familiarity/experience with AI	Risk of bias^a^
Goh, 2024	Several Asia-Pacific Countries	Oct 2022–Jan 2023	23-Item online survey	165 (N/A)	• Gastroenterologists (94%), colorectal surgeons (2%), general surgeons (1%), and other (2%) • Residents (12%), fellows (12%), consultants (35%), senior consultants (34%), and other (7%)	67% had direct exposure to AI at work	Low
Kader, 2022	UK	Oct 2020–Feb 2021	Online survey	104 (N/A)	• Senior consultants (52%), trainees (39%), and endoscopists from nursing background (9%) • Tertiary care (52%), secondary care (33%), and university academic centers (15%)	28% attended formal AI teaching or completed courses	Low
Kochhar, 2021	US	March–April 2020	10-Item online survey; part of a larger survey	165 (N/A)	• Seniors and fellows • Academic, community, or private practice setting • Those involved in AI research were excluded	12% attended formal AI conferences or completed courses	Low
Leggett, 2024	US (80%), international (20%)^b^	May–July 2020	16-Item online survey	374 (3.7%)	• Senior (>10 y in practice) gastroenterologists (48%), gastroenterologists (<10 y in practice) (35%), and GI fellows (17%) • Academic setting (51%), private setting (30%), and from hospital (19%)	7% used AI in clinical practice; 11% involved in developing AI applications	Low
Nazarian, 2023	UK	March–April 2021	Anonymous online 18-item survey	75 (40.3%)	• Gastroenterologists (75%) and surgeons (25%) • Senior consultants (59%), registrars, fellows, or endoscopists from nursing background (41%)	77% familiar with AI	Low
Nehme, 2023	US	Sept 2021–March 2022	10-Item anonymous online survey, performed pre and post implementation	16 (70%)	• Seniors (87%) and juniors (13%) • CADe was activated in 52.1% of the colonoscopies (542/1041) performed during implementation.	Preimplementation: 0% formal training/use 100% aware of AI 56% aware of AI use	Low
				17 (74%)		Post: Among those who activated CADe during colonoscopy	
Schulz, 2023	Asia-Pacific region	Nove 2022–Jan 2023	Online survey	164 (N/A)	• Gastroenterologists (93%) and other (7%) • Consultants or senior consultants (67%): <5 y of experience (23%), 5–10 y (18%), 11–20 y (28%), >20 y (23%), undisclosed (8%) • Public hospitals (67%) and other (33%)	68% had used AI in their work; 80% know other clinicians who use AI	Low
Tham, 2023	Singapore	N/A	35-Item online survey	16 (66.7%)	• All endoscopists (100%) • All consultant-grade (100%)	100% used AI-assisted colonoscopy	Low
Tian, 2022	China	April–June 2020	24-Item online survey	210 (N/A)	• All seniors (100%) • Those reluctant to fill out the questionnaire and those who had never used AI were excluded	100% used AI-assisted colonoscopy	Low
Van der Zander, 2022	Netherlands	April 2020–Aug 2021	Paper questionnaires	80 (N/A)	• Senior gastroenterologists (44%) and GI fellows (56%)	25% experience with AI in GI clinical setting	Low
Van der Zander, 2024	Netherlands	June-September 2021	Online survey and qualitative interviews	23 (N/A)	• Senior gastroenterologists (74%) and GI fellows (26%)	43% with previous AI experience	Low
Wadhwa, 2020	US	Dec 2018–Jan 2019	Online survey	124 (38%)	• Senior attendings (93%) and GI fellows (5%) • Private practice (53%) and academic setting (47%)	N/A	Low
Watkins, 2024							
Survey 1	US	October–November 2022	33-Item online survey	22 (92%)	*N/A*	All respondents participated in a pragmatic CADe (GI Genius) implementation trial prior to surveys	Low
Survey 2			8-Item online survey	21 (88%)			

N/A, not ascertained.

a ROB was evaluated using the JBI critical appraisal checklist (https://jbi.global/sites/default/files/2021-10/Checklist_for_Qualitative_Research.docx).

b South America (7.2%), Asia (7.0%), Europe (4.3%), Africa (1.3%), Australia (0.3%).

Table A5 summarizes details on survey characteristics including development and validation processes, (only one was previously validated)^32^ domains and outcomes and distribution method. Most of the surveys include different types of questions (eg, yes/no, Likert-based). Two studies used a presurvey and postsurvey where an initial baseline assessment was conducted among participants with no experience using AI followed by a postimplementation evaluation among providers who opted to activate CADe during colonoscopy.^28^^,^^36^ In the study by Watkins et al,^36^ authors also conducted semiqualitative 1-to-1 Zoom interviews with some of the participants after informing them of trial results but without allowing participants to discuss with one another. Data were analyzed according to the principles of thematic analysis.

### Positive Attitudes and Perceptions of AI in Colonoscopy

#### Interest and satisfaction in using AI in colonoscopy

**Figure 1:**
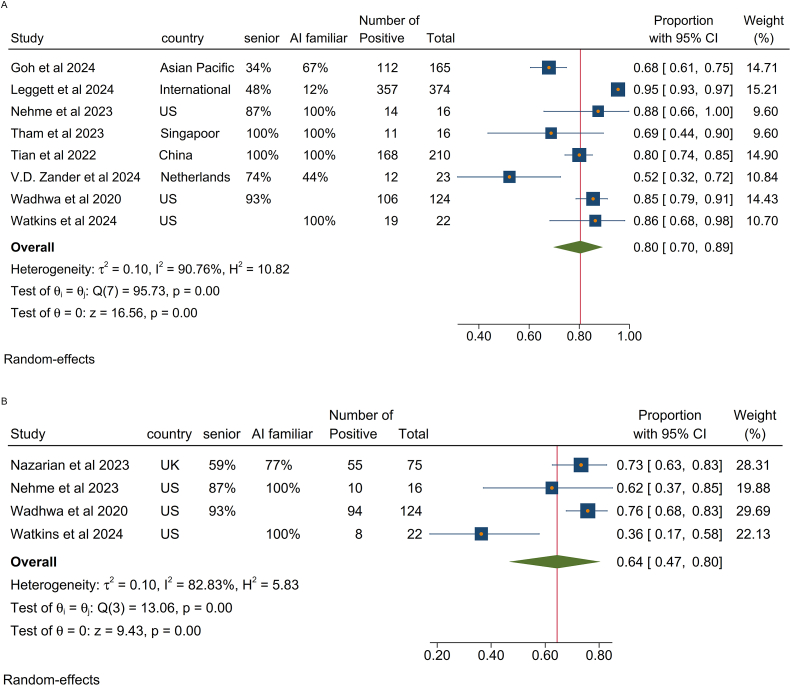
Provider perspective about using AI in colonoscopy. Pp of participants who were interested in using AI in colonoscopy (A) and believed it can improve lesion detection rate (B).

Eight studies including 950 participants^24^^,^^28^^,^^29^^,^^31^, ^32^, ^33^^,^^35^^,^^36^ reported on provider interest and satisfaction with using AI in clinical practice and most studies (n = 5) included senior gastroenterologists with experience or familiarity with AI (Figure 1A). There was a high level of interest or satisfaction in adopting AI for colonoscopy (Pp = 80%, 95% CI: 70%, 89%, *I*^*2*^ = 91%). One outlier study from the Netherlands indicated lower interest among providers toward CADx at 52%.^33^

Among 4 studies with 237 participants^27^^,^^28^^,^^35^^,^^36^ a high proportion of respondents endorsed the potential value of CADe to improve adenoma or polyp detection rate (Pp = 64%, 95% CI: 47%, 80%, *I*^*2*^ = 83%) (Figure 1B). Most included participants from these studies were senior providers. A relatively lower rate of confidence in AI to improve ADR (36%) was seen in a US study that interviewed participants of a pragmatic trial in an academic setting, in which using a CADe platform did not improve adenoma detection.^36^

#### Top perceived advantages of using AI in colonoscopy

The highest ranked perceived advantages (voted by >50% of responders) for using AI in colonoscopy were improving endoscopic diagnosis^25^^,^^27^^,^^28^^,^^32^^,^^35^ and improving quality of endoscopy^25^^,^^26^^,^^31^^,^^34^ (Table A6). In the studies where respondents indicated a high value placed on potential improved efficiency with AI, there were fewer senior participants.

### Perceived Disadvantages or Concerns with Using AI in Colonoscopy

#### Cost of AI

**Figure 2:**
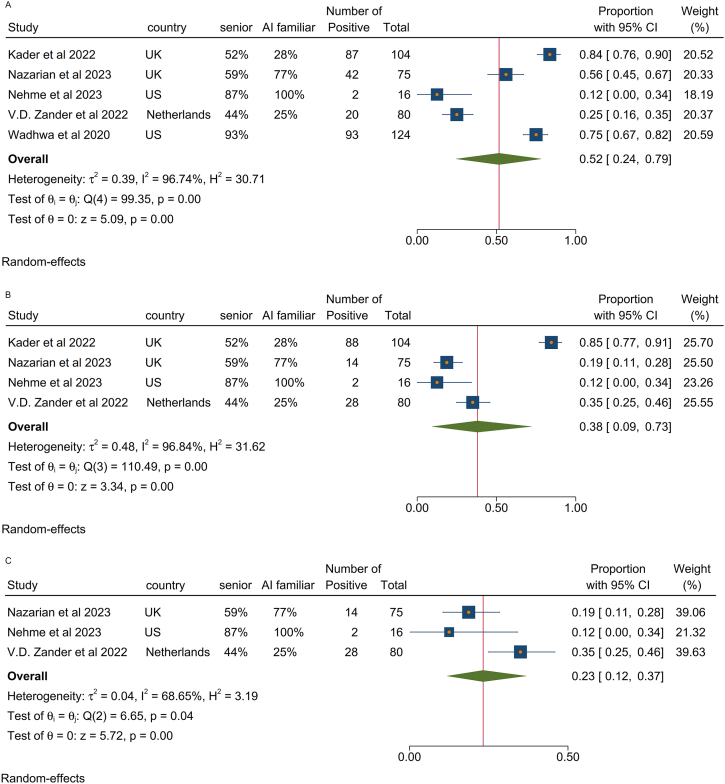
Perceived concerns about using AI in colonoscopy. Pp of participants who were concerned about (A) cost, (B), accountability for misdiagnosis, or (C) medicolegal issues of using AI in colonoscopy.

There were 5 studies (n = 399 respondents) that reported on providers’ perspective regarding financial implications of adopting AI^25^^,^^27^^,^^28^^,^^34^^,^^35^ Approximately half of providers (Pp = 52%, 95% CI: 24%, 79%, *I*^*2*^ = 97%) were concerned about the cost of using AI in colonoscopy (Figure 2A). There was significant heterogeneity among the studies for perceived disadvantage related to cost ranging from 12% in Nehme et al^28^ to 84% in Kader et al.^25^ Both studies were conducted in the US with a similar distribution of senior and junior gastroenterologists. However, Wadhwa et al conducted their study in 2018–2019 and the study by Nehme et al was conducted in 2021–2022. Therefore, time when the study was conducted might be related to the heterogeneity between these 2 studies. Another important source of heterogeneity is the setting in which the study was conducted. Providers at well-funded centers, such as the one in the study by Nehme et al,^28^ may have fewer concerns about the cost of AI, whereas those in resource-limited settings are likely to view cost as a more significant barrier.

Leggett et al included a question to participants about who they think should be responsible for the cost of incorporating AI.^29^ While 46% of the providers from an academic setting thought the hospital should be responsible for the cost, only 28% who were in private practice felt the hospital should be responsible for AI cost. About 27% of the providers from the private practice believed a Current Procedural Terminology code shall be responsible for the cost of AI set up.^29^

#### Accountability of AI for misdiagnosis and medicolegal issues

Four studies^25^^,^^27^^,^^28^^,^^34^ reported on the issue of lack of accountability for misdiagnosis with AI and a total of 132 of 275 respondents raised this concern (Pp = 38%, 95% CI: 9%, 73%, *I*^*2*^ = 97%). (Figure 2B). As indicated by the high *I*^*2*^ index, we observed significant heterogeneity in proportion of the participants who raised this concern among these studies ranging from 12%^28^ to 85%^25^ which may be related to the study characteristics. For example, Kader et al^25^ reported higher rate of accountability concern compared to Van der Zander et al^34^ (85% vs 35%) which may be related to different clinical settings. Participants in Kader et al were from referral and academic centers in UK while those in Van der Zander et al were from different Dutch hospitals. Notably, the participants in both studies had comparable proportion of senior providers and those familiar with AI. Furthermore, comparing Kader et al^25^ with the study by Nazarian et al^27^ who reported lower rate of accountability concern (19% vs 85%) reveals the participants in Nazarian et al have similar country of origin and proportion of senior providers, but have higher proportion of familiarity with AI compared to the participants in Kader et al (77% vs 28%). Lower rate of accountability concern in Nazarian et al study might be related to the fact that higher proportion of responders were familiar with AI.

Furthermore, 3 studies focused on the potential medicolegal concern with AI in colonsocopy.^27^^,^^28^^,^^34^ A low proportion of respondents (Pp = 23%, 95% CI: 12%, 37%, *I*^*2*^ = 69%) indicated hesitation with integrating AI into their clinical practice based on medicolegal issues (Figure 2C).

#### Top perceived disadvantages of using AI in colonoscopy

In 3 studies,^25^^,^^27^^,^^35^ cost was voted among the top ranked perceived disadvantage/concerns of adopting AI in colonoscopy (voted by >50% of participants). Additional issues were cited as a high number of false positives and low diagnosis accuracy^28^^,^^36^ and lack of guidelines related to AI use in colonsocopy^25^^,^^27^ (Table A7). Notably, some studies reported conflicting advantages and disadvantages for AI adoption. For example, 61% of participants in Kochhar et al^26^ felt that AI will increase colonoscopy efficiency but 18% were concerned that providers would be less efficient with patient care with AI.

### Impact of Experience and Clinical Setting on Providers’ Perspective toward AI

Some of the studies compared the outcome variables in different subgroups of participants. Regarding impact of clinical setting, a higher proportion of providers from private practice perceived cost among main challenges for AI adoption compared to providers from academic setting (87.5% vs 63.5%).^35^ Furthermore, higher proportion of academic providers believed CADe can improve endoscopist satisfaction (81% vs 48%). However, academic and private practice providers had overall comparable interest in CADe.^35^

Several studies compared senior providers and junior providers/trainees in terms of their perspective toward AI adoption. In 2 studies from Europe, senior and more experienced providers were more willing to use AI^34^ and more likely to believe CADe can provide solutions for complex care tasks^34^ (31% vs 13%) and improve colonoscopy quality (84% vs 48%), ADR (82% vs 52%), and polyp detection rate (88% vs 62%),^27^ compared to less-experienced providers. Furthermore, high acceptance of CADe was seen in higher proportion of Chief and Associated Chief physicians (82%) compared to other physicians (50%) in a study from China.^32^ Senior providers also expected faster implementation of AI and were more likely to expect workflow change by AI.^34^ In contrast to these studies, senior and junior providers reported comparable interest in AI in an older study conducted in the US. Furthermore, this study reported a higher proportion of junior providers believed CADe would increase ADR (82% vs 71%) and number of polyps removed (91% vs 72%).^35^

On the other hand, a higher proportion of trainees and junior providers considered faster endoscopy^25^ (53% vs 24%) and time saving for physicians^34^ (62% vs 46%) among main advantages of AI implementation compared to senior providers. In addition, they demonstrated lower concern regarding the challenge of staying up to date with AI advances^25^ (66% vs 82%). However, concern for loss of skills by AI was far more prominent among fellows (42%) compared to senior providers^34^ (0%).

### Impact of CADe Implementation and Exposure on Providers’ Perspective

Nehme et al^28^ conducted preimplementation and postimplementation surveys (Table). At baseline, 87.5% of the physicians without a prior experience of using AI were willing to use the technology or were fully embracing it. Also, mean attitude toward AI and mean overall enthusiasm were 3.1 and 7.9, respectively. CADe was available to all the providers during the entire study period and was activated in 52.1% of the colonoscopies (542 of 1041) performed. Based on a postimplementation survey among the providers who decided to activate CADe (GI Genius from Medtronic) during colonoscopy, average overall experience was rated at 6.3 on a 10-point Likert system. Too many false positive signals was the main concern of providers both at baseline (69%) and postimplementation (82%) (Table A7). While 37% and 25% of responders expressed concerns regarding prolonged procedure time and CADe being too distracting at baseline, this increased to 47% and 59% of the providers who activated CADe, respectively. Reassurance nothing was missed was the top perceived (71%) advantage of CADe postimplementation (Table A6). Eventually, 65% of providers who activated CADe believed there is a strong role for AI in colonoscopy, but that AI will need to be further refined.

In another study^36^ that surveyed providers at baseline, after participating in a pragmatic trial to use GI Genius CADe, and after hearing the results of the trial, the proportion of providers who endorsed enthusiasm toward using CADe was 88% at baseline and slightly increased to 90% after participating in the pragmatic trial. After trial, providers thought CADe was easy to use (100%), helped them focus on exposing all the colonic mucosa (48%), and provided comfort as a second pair of eyes (44%). However, they raised concerns about frequent false positive detection (44%) as well as the green boxes and the sound that goes along with it being bothersome (40%–43%).^36^ The authors also conducted semiqualitative interviews with some of the participants who heard the trial results. Key themes for advantages and disadvantages that emerged were in line with postimplementation survey results (eg concerns about the frequent occurrence of false positives, extended procedure duration). Several participants also expressed disappointment when the CADe modules were withdrawn at the end of the pragmatic trial.

### Risk of Bias Assessment

Overall, the studies were rated low for risk of bias (ROB). In 6 studies,^25^, ^26^, ^27^^,^^31^^,^^35^^,^^36^ we had concern regarding one of the 10 JBI checklist items (Table A8). The remaining studies did not have concern for ROB in any of the appraisal survey. Of note, this assessment does not address limitations with the instrument used in the study. Since most of the studies did not use validated surveys, it is possible that the risk of bias was underestimated.

## Discussion

In this review, we summarized the attitudes and perspective of providers with a wide range of experience and familiarity with AI and from different clinical settings toward adopting AI in colonoscopy. Overall, most providers were interested in using AI and believed that it can improve adenoma and polyp detection. Furthermore, improving quality of colonoscopy was among the major perceived advantages of adopting AI. The most common reported barrier was cost and there was an explicit concern regarding the lack of supporting societal and clinical guidelines.

Attitudes toward AI adoption varied by seniority. Senior providers are more willing to use AI and were more likely to believe CADe can improve quality of endoscopy and ADR, with 83% of participants expressing interest in AI and 74% of participants indicating a belief that CADe systems improve ADR.^27^^,^^34^ This might reflect more familiarity with or exposure to these systems compared to trainees or a more comprehensive perspective based on years of experience and insight that recognizes the potential value of this major innovation in colonoscopy and disruptive technology. We note that this difference was not seen in an older study when majority of providers (regardless of experience) were not familiar with AI.^35^ Another factor that can impact how providers view AI is the year when the study was conducted. Earlier studies (eg Wadhwa et al)^35^ were done when only experimental AI platforms were available and much less accepted by not just GI community, but by the public at large. In contrast, later studies were done when Food and Drug Administration–approved platforms were available that could have made positive impact on perspectives toward AI. A subgroup analysis of 2 studies aggregating the views of gastroenterology fellows suggested that fellows believed that CADe will result in faster procedures and time savings.^25^^,^^34^ We also found studies that ranked improved efficiency among top perceived advantages included lower proportion of senior providers. This might be related to the fact that efficiency is more challenging for trainees; therefore, they are more likely to endorse this aspect, whereas senior practicing endoscopists who are likely already efficient are looking for other benefits from AI assisted colonoscopy.

Concerns about AI in colonoscopy varied depending on the year when the study was conducted, suggesting a possible shift as CADe systems were deployed into clinical practice in the United States. While 75% of participants endorsed cost as the main disadvantage in a study conducted from 2018 to 2019, a separate study conducted from 2021 to 2022 found that only 12% of the participants reported this as the main disadvantage.^28^^,^^35^ Both studies are from the US and have a similar proportion of senior providers but the later study had a high proportion (94%) of participants who reported familiarity with AI.^28^ Also with regard to the financial implications, a higher proportion of private practice providers expressed concerns regarding cost of CADe.^35^ The proportion of providers from an academic center who were concerned about accountability for AI misdiagnosis was high (85%) compared to a study focused on private practice providers (35%).^25^^,^^34^ An important factor that can impact providers concern regarding medicolegal issues is the country where the study was conducted. The legal frameworks and liability standards in place in each country (eg the US vs Europe) can impact how much providers are concerned about the medicolegal issues of AI adoption and contribute to heterogeneity among studies.

Studies that addressed postimplementation perspectives toward CADe may reflect important insights from early adopters of the technology. In Nehme et al,^28^ although the survey questions differed across time points, postimplementation experience (6.3) was rated more positively than initial attitudes (3.1) but less favorably than initial enthusiasm (7.9). Concerns about false positives, extended procedure times, and the distracting nature of the CADe increased after implementation. The study by Watkins et al^36^) found that provider enthusiasm was already high at baseline (88%) and increased slightly to 90% after participating in a pragmatic trial using CADe. Differences in software version and proportion of providers with high experience may account for the varied trends between 2 studies. In the semiqualitative and semistructured Zoom interviews described in Watkins et al, thematic analysis revealed concerns about false positives, procedure length, and distractions, and highlighted perceived benefits such as the reassurance of a “second set of eyes,” improved scope stabilization, and a sense of friendly competition in identifying polyps. Some participants even expressed disappointment when the CADe modules were removed at the end of the trial. Further studies are needed that use mixed methods and focus groups to gain insight into user experience."

### Applicability of Our Findings

In 2 studies, the absence of clinical guidelines for AI systems in colonoscopy was expressed as a major concern for AI integration (92%^25^ of participants expressed and in another study by 51%^27^ of the participants). Thos unmet need is now being addressed with the clinical guidelines for AI assisted colonoscopy by the AGA and BMJ Rapid Recommendations.^37^^,^^38^ This paper provides critical and meaningful data on endoscopists’ attitudes, perspective, perceived facilitators and barriers toward AI assisted colonoscopy overall and by important subgroups. This information helped direct the guideline development process and drove tailored recommendations that take into account the concerns and desires of providers as an overall strategy to facilitate implementation and adoption of recommendations. Furthermore, this review highlighted crucial gaps in the literature that can guide future research to central questions, such as cost, medicolegal aspects of adopting CADe, and false positives to provide evidence that can help providers address their concerns and hesitations regarding CADe applicability.

This systematic and literature review summarizes studies that surveyed providers about their perspective toward using AI in colonoscopy. Data synthesis based on a large, pooled sample size of more than 1500 participants provides more accurate insights on different aspects of perspective toward using AI. Lack of a standardized questionnaire is a major limitation for the generalizability of findings in individual studies and poses a challenge for pooling the evidence for providers’ perspective toward AI. Significant heterogeneity among studies in terms of country of origin, clinical setting, proportion of senior providers and those familiar with AI make it challenging to reach a conclusion on participants’ attitude. Finally, lower overall sample size of available studies limits the robustness in available findings.

Notably, most of the surveys used were not validated instruments, and it is certainly possible that the question format and answer choices influenced the response. Furthermore, most of the studies did not mention a specific AI platform and only 3 studies^28^^,^^31^^,^^36^ evaluated providers experience with the same AI platform (GI Genius). Type of AI platform may influence users’ opinion about cost-effectiveness, false positive rate, and user-friendliness. Since AI software is constantly evolving and updating its performance characteristics, variability within the same platform over time and AI software fluidity should be evaluated over time.

## Conclusion

Most providers with different backgrounds, level of expertise, and familiarity with AI endorsed interest in adopting AI in colonoscopy and believed it can improve diagnostic accuracy. However, providers expressed major concerns regarding cost, responsibility for misdiagnosis, and medicolegal issues. Providers also expressed a need for clinical guidelines from professional societies on how to adopt AI in their routine practice. These findings help inform clinical guidelines and innovation that incorporate endoscopist values and preferences.

## Supplementary materials

Tables A1–A8 and Figure A1
