# Supplementary material for: Provider Attitudes and Perceptions on Using Artificial Intelligence in Colonoscopy: A Systematic Review and Meta-Analysis
Source: Gastro Hep Adv. 2025 Jul 11;4(10):100746. doi: 10.1016/j.gastha.2025.100746 (PMC12410009; doi:10.1016/j.gastha.2025.100746)
Supplement: Tables A1–A8 and Figure A1 [file mmc1.docx]

**Supplemental Table 1: Reporting Guideline Checklists**

PRISMA 2020 Main Checklist

| **Topic** | **No.** | **Item** | **Location where item is reported** |
| --- | --- | --- | --- |
| **TITLE** |  |  |  |
| **Title** | 1 | Identify the report as a systematic review. | LN |
| **ABSTRACT** |  |  |  |
| **Abstract** | 2 | See the PRISMA 2020 for Abstracts checklist | Suppl. |
| **INTRODUCTION** |  |  |  |
| **Rationale** | 3 | Describe the rationale for the review in the context of existing knowledge. | LN |
| **Objectives** | 4 | Provide an explicit statement of the objective(s) or question(s) the review addresses. | LN |
| **METHODS** |  |  |  |
| **Eligibility criteria** | 5 | Specify the inclusion and exclusion criteria for the review and how studies were grouped for the syntheses. | LN |
| **Information sources** | 6 | Specify all databases, registers, websites, organisations, reference lists and other sources searched or consulted to identify studies. Specify the date when each source was last searched or consulted. | LN |
| **Search strategy** | 7 | Present the full search strategies for all databases, registers and websites, including any filters and limits used. | LN |
| **Selection process** | 8 | Specify the methods used to decide whether a study met the inclusion criteria of the review, including how many reviewers screened each record and each report retrieved, whether they worked independently, and if applicable, details of automation tools used in the process. | LN |
| **Data collection process** | 9 | Specify the methods used to collect data from reports, including how many reviewers collected data from each report, whether they worked independently, any processes for obtaining or confirming data from study investigators, and if applicable, details of automation tools used in the process. | LN |
| **Data items** | 10a | List and define all outcomes for which data were sought. Specify whether all results that were compatible with each outcome domain in each study were sought (e.g. for all measures, time points, analyses), and if not, the methods used to decide which results to collect. | LN |
|  | 10b | List and define all other variables for which data were sought (e.g. participant and intervention characteristics, funding sources). Describe any assumptions made about any missing or unclear information. | LN |
| **Study risk of bias assessment** | 11 | Specify the methods used to assess risk of bias in the included studies, including details of the tool(s) used, how many reviewers assessed each study and whether they worked independently, and if applicable, details of automation tools used in the process. | LN |
| **Effect measures** | 12 | Specify for each outcome the effect measure(s) (e.g. risk ratio, mean difference) used in the synthesis or presentation of results. | LN |
| **Synthesis methods** | 13a | Describe the processes used to decide which studies were eligible for each synthesis (e.g. tabulating the study intervention characteristics and comparing against the planned groups for each synthesis (item 5)). | LN |
|  | 13b | Describe any methods required to prepare the data for presentation or synthesis, such as handling of missing summary statistics, or data conversions. | LN |
|  | 13c | Describe any methods used to tabulate or visually display results of individual studies and syntheses. | LN |
|  | 13d | Describe any methods used to synthesize results and provide a rationale for the choice(s). If meta-analysis was performed, describe the model(s), method(s) to identify the presence and extent of statistical heterogeneity, and software package(s) used. | LN |
|  | 13e | Describe any methods used to explore possible causes of heterogeneity among study results (e.g. subgroup analysis, meta-regression). | LN |
|  | 13f | Describe any sensitivity analyses conducted to assess robustness of the synthesized results. | LN |
| **Reporting bias assessment** | 14 | Describe any methods used to assess risk of bias due to missing results in a synthesis (arising from reporting biases). | LN |
| **Certainty assessment** | 15 | Describe any methods used to assess certainty (or confidence) in the body of evidence for an outcome. | LN |
| **RESULTS** |  |  |  |
| **Study selection** | 16a | Describe the results of the search and selection process, from the number of records identified in the search to the number of studies included in the review, ideally using a flow diagram. | LN |
|  | 16b | Cite studies that might appear to meet the inclusion criteria, but which were excluded, and explain why they were excluded. | LN |
| **Study characteristics** | 17 | Cite each included study and present its characteristics. | LN |
| **Risk of bias in studies** | 18 | Present assessments of risk of bias for each included study. | LN |
| **Results of individual studies** | 19 | For all outcomes, present, for each study: (a) summary statistics for each group (where appropriate) and (b) an effect estimate and its precision (e.g. confidence/credible interval), ideally using structured tables or plots. | LN |
| **Results of syntheses** | 20a | For each synthesis, briefly summarise the characteristics and risk of bias among contributing studies. | LN |
|  | 20b | Present results of all statistical syntheses conducted. If meta-analysis was done, present for each the summary estimate and its precision (e.g. confidence/credible interval) and measures of statistical heterogeneity. If comparing groups, describe the direction of the effect. | LN |
|  | 20c | Present results of all investigations of possible causes of heterogeneity among study results. | LN |
|  | 20d | Present results of all sensitivity analyses conducted to assess the robustness of the synthesized results. | LN |
| **Reporting biases** | 21 | Present assessments of risk of bias due to missing results (arising from reporting biases) for each synthesis assessed. | LN |
| **Certainty of evidence** | 22 | Present assessments of certainty (or confidence) in the body of evidence for each outcome assessed. | LN |
| **DISCUSSION** |  |  |  |
| **Discussion** | 23a | Provide a general interpretation of the results in the context of other evidence. | LN |
|  | 23b | Discuss any limitations of the evidence included in the review. | LN |
|  | 23c | Discuss any limitations of the review processes used. | LN |
|  | 23d | Discuss implications of the results for practice, policy, and future research. | LN |
| **OTHER INFORMATION** |  |  |  |
| **Registration and protocol** | 24a | Provide registration information for the review, including register name and registration number, or state that the review was not registered. | LN |
|  | 24b | Indicate where the review protocol can be accessed, or state that a protocol was not prepared. | LN |
|  | 24c | Describe and explain any amendments to information provided at registration or in the protocol. | LN |
| **Support** | 25 | Describe sources of financial or non-financial support for the review, and the role of the funders or sponsors in the review. | LN |
| **Competing interests** | 26 | Declare any competing interests of review authors. | LN |
| **Availability of data, code and other materials** | 27 | Report which of the following are publicly available and where they can be found: template data collection forms; data extracted from included studies; data used for all analyses; analytic code; any other materials used in the review. | LN |

From: Page MJ, McKenzie JE, Bossuyt PM, Boutron I, Hoffmann TC, Mulrow CD, et al. The PRISMA 2020 statement: an updated guideline for reporting systematic reviews. MetaArXiv. 2020, September 14. DOI: 10.31222/osf.io/v7gm2. For more information, visit: www.prisma-statement.org

# PRISMA Abstract Checklist

| **Topic** | **No.** | **Item** | **Reported?** |
| --- | --- | --- | --- |
| **TITLE** |  |  |  |
| **Title** | 1 | Identify the report as a systematic review. | Yes |
| **BACKGROUND** |  |  |  |
| **Objectives** | 2 | Provide an explicit statement of the main objective(s) or question(s) the review addresses. | Yes |
| **METHODS** |  |  |  |
| **Eligibility criteria** | 3 | Specify the inclusion and exclusion criteria for the review. | Yes |
| **Information sources** | 4 | Specify the information sources (e.g. databases, registers) used to identify studies and the date when each was last searched. | Yes |
| **Risk of bias** | 5 | Specify the methods used to assess risk of bias in the included studies. | Yes |
| **Synthesis of results** | 6 | Specify the methods used to present and synthesize results. | Yes |
| **RESULTS** |  |  |  |
| **Included studies** | 7 | Give the total number of included studies and participants and summarise relevant characteristics of studies. | Yes |
| **Synthesis of results** | 8 | Present results for main outcomes, preferably indicating the number of included studies and participants for each. If meta-analysis was done, report the summary estimate and confidence/credible interval. If comparing groups, indicate the direction of the effect (i.e. which group is favoured). | Yes |
| **DISCUSSION** |  |  |  |
| **Limitations of evidence** | 9 | Provide a brief summary of the limitations of the evidence included in the review (e.g. study risk of bias, inconsistency and imprecision). | Yes |
| **Interpretation** | 10 | Provide a general interpretation of the results and important implications. | Yes |
| **OTHER** |  |  |  |
| **Funding** | 11 | Specify the primary source of funding for the review. | Yes |
| **Registration** | 12 | Provide the register name and registration number. | Yes |

**Supplemental Table 2: Search Strategies**

**Ovid Embase**

1 exp artificial intelligence/

2 deep learning/

3 exp computer assisted diagnosis/

4 ((artificial or computat* or computer* or machine or deep or transfer or hierarchical) adj1 (intelligence* or learning* or reasoning*)).tw,kf.

5 (neural network* or random forest* or decision tree* or knowledge representation* or computer vision system* or computer reasoning* or natural language processing or perceptron* or connectionist model* or expert system* or computer assisted).tw,kf.

6 AI.ti,ab.

7 or/1-6

8 exp colonoscopy/

9 (colonscop* or coloscop* or colonoscop* or ileocolonoscop* or sigmoidoscop* or proctosigmoidoscop*).tw,kf.

10 (colon* adj3 endoscop*).tw,kf.

11 8 or 9 or 10

12 7 and 11

13 exp trust/

14 (accept* or confidence or dislik* or distrust* or fear* or hesita* or mistrust* or percept* reluctan* or refus* or trust* or willing*).tw,kf.

15 13 or 14

16 12 and 15

**Ovid MEDLINE(R) ALL**

1 exp artificial intelligence/

2 exp Diagnosis, Computer-Assisted/

3 ((artificial or computat* or computer* or machine or deep or transfer or hierarchical) adj1 (intelligence* or learning* or reasoning*)).tw,kf.

4 (neural network* or random forest* or decision tree* or knowledge representation* or computer vision system* or computer reasoning* or natural language processing or perceptron* or connectionist model* or expert system* or computer assisted).tw,kf.

5 AI.ti,ab.

6 or/1-5

7 exp Colonoscopy/

8 (colonscop* or coloscop* or colonoscop* or ileocolonoscop* or sigmoidoscop* or proctosigmoidoscop*).tw,kf.

9 (colon* adj3 endoscop*).tw,kf.

10 7 or 8 or 9

11 6 and 10

12 exp trust/

13 (accept* or confidence or dislik* or distrust* or fear* or hesita* or mistrust* or percept* or reluctan* or refus* or trust* or willing*).tw,kf.

14 12 or 13

15 11 and 14

**Scopus**

( TITLE-ABS-KEY ( accept* OR confidence OR dislik* OR distrust* OR fear* OR hesita* OR mistrust* OR percept* OR reluctan* OR refus* OR trust* OR willing* ) ) AND ( TITLE-ABS-KEY ( colonscop* OR coloscop* OR colonoscop* OR ileocolonoscop* OR sigmoidoscop* OR proctosigmoidoscop* ) OR TITLE-ABS-KEY ( colon* W/3 endoscop* ) ) AND ( TITLE-ABS-KEY ( ( artificial OR computat* OR computer* OR machine OR deep OR transfer OR hierarchical ) W/1 ( intelligence* OR learning* OR reasoning* ) ) OR TITLE-ABS-KEY ( ai OR "neural network*" OR "random forest*" OR "decision tree*" OR "knowledge representation*" OR "computer vision system*" OR "computer reasoning*" OR "natural language processing" OR perceptron* OR "connectionist model*" OR "expert system*" OR "computer assisted" ) )

**Web of Science Core Collection**

#1 TS=((artificial or computat* or computer* or machine or deep or transfer or hierarchical) near/1 (intelligence* or learning* or reasoning*)) or TS=(AI or "neural network*" or "random forest*" or "decision tree*" or "knowledge representation*" or "computer vision system*" or "computer reasoning*" or "natural language processing" or perceptron* or "connectionist model*" or "expert system*" or "computer assisted")

#2 TS=(colonscop* or coloscop* or colonoscop* or ileocolonoscop* or sigmoidoscop* or proctosigmoidoscop*) or TS=(colon* near/3 endoscop*)

#3 TS=(accept* or confidence or dislik* or distrust* or fear* or hesita* or mistrust* or percept* or reluctan* or refus* or trust* or willing*)

#4 #1 and #2 and #3

The Core Collection included in this review is:

1. Science Citation Index Expanded (1900 - Data Searched)

2. Social Sciences Citation Index (1900 - Date Searched)

3. Art & Humanities - (1975 - Date Searched)

4. Conference Proceedings Citation Index - Science (1991 - Date Searched)

5. Conference Proceedings Citation Index - Social Sciences and Humanities (1991 - Date Searched)

6. Book Citation Index - Science (2005 - Date Searched)

7. Book Citation Index - Social Sciences and Humanities ( 2005 - Date Searched)

8. Emerging Source Citation Index - (2018 - Date Searched)

9. Current Chemical Reactions (1985 - Date Searched)

10. Index Chemicus (1993 - Date Searched)

**Cochrane Library**

#1 ((artificial or computat* or computer* or machine or deep or transfer or hierarchical) near/3 (intelligence* or learning* or reasoning*)):ti,ab or (neural network* or random forest* or decision tree* or knowledge representation* or computer vision system* or computer reasoning* or natural language processing or perceptron* or connectionist model* or expert system* or computer assist*):ti,ab

#2 (colonscop* or coloscop* or colonoscop* or ileocolonoscop* or sigmoidoscop* or proctosigmoidoscop*):ti,ab or (colon* near/3 endoscop*):ti,ab

#3 (accept* or confidence or dislik* or distrust* or fear* or hesita* or mistrust* or percept* or reluctan* or refus* or trust* or willing*):ti,ab

#4 #1 and #2 and #3

The Cochrane Library database includes:

1. Cochrane Database of Systematic Reviews

2. Cochrane Central Register of Controlled Trials

3. Cochrane Clinical Answers

**PubMed**

(((accept*[Title/Abstract] OR confidence[Title/Abstract] OR dislik*[Title/Abstract] OR distrust*[Title/Abstract] OR fear*[Title/Abstract] OR hesita*[Title/Abstract] OR mistrust*[Title/Abstract] OR percept*[Title/Abstract] OR reluctan*[Title/Abstract] OR refus*[Title/Abstract] OR trust*[Title/Abstract] OR willing*[Title/Abstract])) AND (colon* endoscop*[Title/Abstract] OR colonscop*[Title/Abstract] OR coloscop*[Title/Abstract] OR colonoscop*[Title/Abstract] OR ileocolonoscop*[Title/Abstract] OR sigmoidoscop*[Title/Abstract] OR proctosigmoidoscop*[Title/Abstract])) AND (((neural network*[Title/Abstract] OR random forest*[Title/Abstract] OR decision tree*[Title/Abstract] OR knowledge representation*[Title/Abstract] OR computer vision system*[Title/Abstract] OR computer reasoning*[Title/Abstract] OR natural language processing[Title/Abstract] OR perceptron*[Title/Abstract] OR connectionist model*[Title/Abstract] OR expert system*[Title/Abstract] OR computer assist*[Title/Abstract]) OR (AI[Title/Abstract]) OR (Artificial intelligence*[Title/Abstract] OR computat* intelligence*[Title/Abstract] OR computer* intelligence*[Title/Abstract] OR machine intelligence*[Title/Abstract] OR deep intelligence*[Title/Abstract] OR transfer intelligence*[Title/Abstract] OR hierarchical intelligence*[Title/Abstract] OR artificial learning*[Title/Abstract] OR computat* learning*[Title/Abstract] OR computer* learning*[Title/Abstract] OR machine learning*[Title/Abstract] OR deep learning*or transfer learning*[Title/Abstract] OR hierarchical learning*[Title/Abstract] OR artificial reasoning*[Title/Abstract] OR computat* reasoning*[Title/Abstract] OR computer* reasoning*[Title/Abstract] OR machine reasoning*[Title/Abstract] OR deep reasoning*[Title/Abstract] OR transfer reasoning*[Title/Abstract] OR hierarchical reasoning*[Title/Abstract]))

**Google Scholar (via Harzing’s Publish or Perish Edition: 8.7.4245.8399)**

artificial intelligence colonoscopy trust

**Supplemental Table 3: Excluded Studies Table**

| **Studies Excluded from the Search Results** | | | | |
| --- | --- | --- | --- | --- |
| **First Authors Last Name** | **Year** | **Title** | **Journal** | **Reason for Exclusion** |
| Ahmad | 2021 | Early evaluation of a computer assisted polyp detection system in bowel cancer screening | Gut | Conference abstract |
| Ahmad | 2021 | Establishing key research questions for the implementation of artificial intelligence in colonoscopy: a modified Delphi method | Endoscopy | Wrong study design |
| Ameen | 2022 | Improving colorectal cancer screening-consumer-centred technological interventions to enhance engagement and participation amongst diverse cohorts | Clinics and research in hepatology and gastroenterology | No original research |
| Anonymous | 2018 | Towards trustable machine learning | Nature | No original research |
| Antes | 2021 | Exploring perceptions of healthcare technologies enabled by artificial intelligence: an online, scenario-based survey | BMC Medical Informatics and Decision Making | Wrong population |
| BinGoh | 2024 | Senior Endoscopists Are More Likely to Trust and Accept Ai-Assisted Colonoscopy for Detection and Treatment of Polyps Compared to Junior Endoscopists | Gastrointestinal Endoscopy | Conference abstract |
| Coronel | 2022 | Physician and Staff Attitudes Towards Implementation of Artificial Intelligence-Assisted Colonoscopy | Gastroenterology | Conference abstract |
| Dolwani | 2019 | Feasibility and accept ability of novel colonoscopy with computer aided early diagnosis of bowel cancer | United European Gastroenterology Journal | Duplicate data |
| Dolwani | 2019 | Feasibility and acceptability of novel colonoscopy with computer aided early diagnosis of bowel cancer | Gut | Conference abstract |
| Goh | 2024 | SENIOR ENDOSCOPISTS ARE MORE LIKELY TO TRUST AND ACCEPT AI-ASSISTED COLONOSCOPY FOR DETECTION AND TREATMENT OF POLYPS | Gastrointestinal Endoscopy | Duplicate data |
| Hicks | 2024 | Visual explanations for polyp detection: How medical doctors assess intrinsic versus extrinsic explanations | PLoS ONE | Wrong outcomes |
| Imperiale | 2011 | Provider acceptance, safety, and effectiveness of a computer-based decision tool for colonoscopy preparation | International Journal of Medical Informatics | Wrong intervention |
| Kader | 2021 | Opinions of uk gastroenterology consultants in the application of artificial intelligence in endoscopy | Gut | Duplicate data |
| Ladabaum | 2023 | Beliefs and Attitudes About Artificial Intelligence (Ai) among Colonoscopist Participants in a Pragmatic Implementation Trial of Computer-Aided Detection (Cade) of Polyps That Did Not Replicate the Positive Results of Randomized Trials | Gastrointestinal Endoscopy | Conference abstract |
| Lee | 2024 | Exploring Endoscopist Perceptions of Artificial Intelligence-Aided Colonoscopy: A Qualitative Analysis | Journal of the Canadian Association of Gastroenterology | Conference abstract |
| Mori | 2024 | Enhancing artificial intelligence-doctor collaboration for computer-aided diagnosis in colonoscopy through improved digital literacy | Digestive Liver Disease | Wrong study design |
| Nigam | 2020 | S0678 A Survey of Knowledge, Perceptions, and Barriers on the Use of Artificial Intelligence in Patients With Inflammatory Bowel Disease in the US and UK | American Journal of Gastroenterology | Duplicate data |
| Nigam | 2021 | PMO-10 Survey on the use of artificial intelligence in IBD patients in the USA and UK | Gut | Conference abstract |
| Nigam | 2021 | Clinician perspectives on the use of artificial intelligence in inflammatory bowel disease | Gastrointestinal Endoscopy | Wrong population |
| Nigam | 2020 | A Survey of Knowledge, Perceptions, and Barriers on the Use of Artificial Intelligence in Patients with Inflammatory Bowel Disease in the U.S. and U.K | American Journal of Gastroenterology | Wrong indication |
| Reverberi | 2022 | Experimental evidence of effective human-AI collaboration in medical decision-making | Scientitic Reports | Wrong outcomes |
| Reverberi | 2021 | Human-cadx interaction | Scientitic Reports | Wrong study design |
| Roumans | 2022 | Appropriate Trust in AI: The Influence of Presenting Different Computer-Aided Diagnosis Variations on the Advice Utilization, Decision Certainty, and Diagnostic Accuracy of Medical Doctors | Thesis | Duplicate data |
| Schmidt | 2024 | Patient Perspectives and Acceptability of Artificial Intelligence Used during Screening Colonoscopy | Gastroenterology | Conference abstract |
| Sinagra | 2021 | Use of Artificial Intelligence in Endoscopic Training: Is Deskilling a Real Fear? | Gastroenterology | Conference abstract |
| Thoufeeq | 2022 | Attitude of Colonoscopists Towards Artificial Intelligence- a Multinational Study | Gut | Conference abstract |
| Thoufeeq | 2022 | P305 Attitude of colonoscopists towards artificial intelligence-a multinational study | Gut | Duplicate data |
| Wadhwa | 2019 | Gastroenterologist sentiment toward artificial intelligence (AI) in endoscopic practice: A nationwide survey | American Journal of Gastroenterology | Duplicate data |
| Winters | 2023 | P233 Barriers and facilitators to colonoscopy in patients and endoscopists | Gut | Duplicate data |
| Winters | 2023 | Patient and Endoscopist Perceptions of New Technologies in Colonoscopy | Gut | Wrong outcomes |
| **Studies Excluded from the Search Results** | | | | |
| **First Authors Last Name** | **Year** | **Title** | **Journal** | **Reason for Exclusion** |
| Areia | 2022 | Cost-effectiveness of artificial intelligence for screening colonoscopy: a modelling study | Lancet Digital Health | Wrong outcomes |
| Ayorinde | 2023 | Healthcare Professionals' Experience of Using Artificial Intelligence: a Systematic Review (Preprint) | Preprint | Wrong study design |
| Botha | 2023 | Artificial Intelligent Tools: Evidence-Mapping on the Perceived Positive Effects on Patient-Care | Preprint | Wrong study design |
| Botha | 2023 | Artificial Intelligence in Healthcare: A Scoping Review of Perceived Threats to Patient Rights and Safety | Preprint | Wrong study design |
| Hummelsberger | 2023 | Insights on the Current State and Future Outlook of AI in Health Care: Expert Interview Study (Preprint) | Preprint | Wrong population |
| Watkins | 2024 | Exploring human–artificial intelligence interactions in a negative pragmatic trial of computer-aided polyp detection | iGIE | Duplicate data |

| **Supplemental Table 4.** Additional Characteristics of studies and participants | | | | | | | |
| --- | --- | --- | --- | --- | --- | --- | --- |
| Study | Participants | | | | | | Study |
|  | **Age (year)** | **Gender** | **AI familiarity** | **Years of experience** | **Number of colonoscopies** | **Average ADR** | **AI platform used** |
| Goh et al 2024 | 44.5 (SD 9.6) | Male 70%  Female 30% | Basic familiarity with AI: 97%  Directly exposed at work: 67% | N/A | N/A | N/A | N/A |
| Kader et al  2022 | N/A | N/A | No formal education or  qualification in AI: 72%  Attendance at an organized AI teaching day: 25%  Completion of an AI course with certification: 3%  Familiar with research methodology in AI:  Not at all: 26%  Slightly: 47%  Moderately: 19%  Very familiar: 8%  AI-related papers read:  None: 15%  < 5: 45%  5–20: 25%  >20: 14% | < 5: 31% (clinical endoscopists and registrars)  > 5: 69%  > 10: 43% | N/A | N/A | N/A |
| Kochhar et al 2021 | N/A | N/A | N/A | N/A | N/A | N/A | N/A |
| Leggett et al 2024 | 46.0 (SD 12.0) | Male: 80% | Currently using AI solutions in practice: 7%  Planning to use AI solutions in the future: 24%  Involved in AI-related research: 11%  Involved in testing AI products and/or solutions: 11% Involved in developing AI applications: 12% | >10: 48%  ≤10: 35%  Fellow: 17% | N/A | N/A | N/A |
| Nazarian et al 2023 | N/A | N/A | N/A | N/A | 0-200: 32%  200-500: 16%  500-1000: 5%  >1000: 47% | 0-15%: 9%  15-25%: 17%  25-40%: 20%  40-60%: 15%  >60%: 7%  Unsure: 32% | N/A |
| Nehme et al 2023 | N/A | Male: 55%  Female: 45% | No clue: 3%  Aware of AI: 47%  Aware of AI and its use: 44%  Well versed in AI: 6%  Formal training: 0% | ≤ 5: 12%  6-10: 13%  11-20: 44%  >20: 31% | N/A | > 45%: 65%  ≤ 45%: 35% | GI Genius |
| Schulz et al 2023 | Mean 44.0 (SD 9.4) | Male: 65%  Female: 23%  Undisclosed: 12% | Used AI in work:  Yes 68%  No 31%  Undisclosed 1% | <5: 23%  6-10: 18%  11-20: 28%  >20: 23%  Undisclosed: 7% | N/A | N/A | N/A |
| Tham et al 2023 | N/A | N/A | N/A | N/A | N/A | N/A | GI Genius |
| Tian et al 2022 | 18-25: 1%  26-40: 64%  41-65: 34%  ≥66: 1% | Male: 58%  Female: 42% | Number of endoscopies with AI:  1-50: 20%  51-100: 26%  101-300: 26%  301-500: 12%  >500: 16% | Chief physician or associate chief physician: 47%  Physician-in-charge 44%  Physician: 11% | N/A | N/A | Endoangel |
| Van Der Zander et al 2022 | Seniors: 49.7 (SD 7.6)  Fellows 32.7 (SD 2.9) | Seniors:  Female 37%  Male 63%  Fellows:  Female 73%  Male 27% | Experience with AI in clinical work:  Seniors: 37%  Fellows: 16% | Years of education among fellows:  2: 2%  3: 42%  4: 22%  5: 21%  6: 13% | N/A | N/A | N/A |
| Van Der Zander et al 2024 | N/A | N/A | N/A | Seniors: 74%  Fellows: 26% | Seniors: 5759  Fellows: 370 |  |  |
| Wadhwa et al 2020 | N/A | N/A | Early adopters for new technologies in gastroenterology: 57% | >15: 55%  <15 years: 35%  Fellow: 10% | Colonoscopy per week:  0-20: 38%  21-40: 45%  41-60: 13%  >60: 2%  No response: 1% | 0-20%: 3%  21-40%: 53%  41-60%: 29%  >60%: 3%  No response: 11% | N/A |
| Watkins et al 2024 | N/A | N/A | N/A | Seniors: 100%  Fellows: 0% | N/A | N/A | GI Genius |

AI: artificial intelligence; GI: Gastroenterology; N/A: not ascertained, CADe: computer-assisted detection

| **Supplemental Table 5.** Characteristics of surveys used in each study | | | | | | |
| --- | --- | --- | --- | --- | --- | --- |
| **Study** | Survey Characteristics | | | | | |
|  | **Validated** | **Structured** | **Development process/workflow** | **Primary domains/outcomes** | **Question types** | **Distribution** |
| Goh et al 2024 | Some items from prior validated survey | Yes | - Used expectancy-value framework, major constructs of the Theory of Planned Behavior  research framework, and the Technology Acceptance Model measures  - Unidimensionality and reliability were verified and assured | Risk perception, acceptance, and trust | -Yes/No  -7-point Likert  -scenario-based | Web-paged |
| Kader et al  2022 | No | No | - A focus group of 5 experts in the field of AI in gastroenterology. Themes and question items were through consensus among members.  -Junior and senior gastroenterologists undertook pilot testing at a teaching hospital in London | Experience in AI, benefits and barriers of adopting AI  in clinical practice, Priorities of and barriers to  research in AI, and priorities for the BSG AI Task  Force | -3-point and 5-point Likert scales  -Multiple-choice | Online newsletter, email, webpage, colleague referral |
| Kochhar et al 2021 | No | No | - Survey was designed using the Survey-Monkey platform (San Mateo, CA, USA  - Before the distribution of the ﬁnal survey to study participants, multiple drafts of the survey were tested by all authors | Most interesting AI applications, main concerns for AI integration into clinical practice, and anticipated startup cost | - Multiple-choice | Online link shared with email and social media platforms (Twitter and LinkedIn) |
| Leggett et al 2024 | No | No | - Designed by the ASGE AI Task Force through a focus-group of 6 gastroenterologists (national and international) with experience in the use and development of AI solutions.  - Iterative editing and committee approval | Perceptions of the current and future implications of AI to the field of Gastroenterology | - Multiple choice  - Yes/No  -Single choice | A web link for survey participation using electronic communication to all ASGE members |
| Nazarian et al 2023 | No | No | - Qualtrics survey software was used |  | - Yes/No  - 4-point Likert scale  -Ranking question  -Multiple-response items | Online through email |
| Nehme et al 2023 | No | No | Pre-implementation and post CADe implementation surveys used. | Pre-implementation: Perceived advantages and disadvantages of AI-assisted colonoscopy, Willingness to implement AI in endoscopy, expected impact of on polyp detection, and procedure times  Post-implementation: experiences and attitudes toward CADe | - Yes/No  -Multi-choice  -10-scale Likert | Online |
| Schulz et al 2023 | No | No | - First pretested the survey with two gastroenterologists. Feedback was gathered from subjects and improvements to the final survey were made using input.  - Based on the expectancy-value framework, major constructs of the TPB research framework, and the TAM measures.  -Some items from prior validated survey | General expectations, attitude, trust, belief and acceptance | -7-scale Likert | Email or WeChat (if they were in China; Qualtrics link |
| Tham et al 2023 | No | No | - Parts of the survey were  modelled based on a prior study  - Survey was conducted two months after using AI-assisted colonoscopy. | Knowledge, perceptions, and behaviors regarding AI-assisted colonoscopy | - Likert 5-scale | Online |
| Tian et al 2022 | Yes | Yes | - Distributed questionnaires to endoscopists who have mainly used ENDOANGEL  -Validated the survey | -Acceptance and satisfaction | - Likert-type | Online |
| Van Der Zander et al 2022 | No | No | - Developed according to the checklist for reporting of survey studies after reviewing literature | Perspective, defined as knowledge, experience, and opinion | -Yes/No;  -5-point Likert  -Multiple response | Self-assessed, paper-based |
| Van Der Zander et al 2024 | No | No |  |  |  |  |
| Wadhwa et al 2020 | No | No |  |  | -Mixed of question types | Online via email |
| Watkins et al 2024 | No | No | - The surveys were adapted from the previously developed “Survey on the Future of Technology-Assisted Work” by social scientists.  - Survey 1 after participations in a trial to evaluate CADe, Survey 2 after hearing the results, qualitative interview | Survey 1: Attitudes and beliefs about CADe  Survey 2: reactions to and explanations for the CADe pragmatic trial results  Qualitative interview: themes understanding and reactions | - 5-point Likert scale  -True/False  -Open ended questions | Online surveys, one-to-one qualitative interviews via Zoom |

AI: artificial intelligence; GI: Gastroenterology; N/A: not ascertained, CADe: computer-assisted detection

**Supplemental Table 6.** Top perceived advantages for adopting AI-assisted colonoscopy

| **Study** | **Rank** | | | | |
| --- | --- | --- | --- | --- | --- |
|  | 1 | 2 | 3 | 4 | 5 |
| **Kader et al 2022** | Quality improvement in endoscopy (97%) | Better endoscopic diagnosis (92%) | Automated reporting (73%) | Fewer clinic appointments (40%) | Shorter endoscopy time (39%) |
| **Kochar et al 2021** | Improve healthcare quality (69%) | Improve efficiency (61%) |  |  |  |
| **Leggett et al 2024** | Provide positive impact on the field of GI (95.5%) | Ease the burden of the EMR (53%) |  |  |  |
| **Nazarian et al 2023** | Increase ADR (73%) | Increase PDR (60%) | Increase confidence (53%) | Training tool (41%) | Productivity (23%) |
| **Nehme et al 2023** | Improving ADR (62%) [Pre-implementation]  Reassurance nothing was missed (71%)  [Post-implementation] | Detecting polyps that could have been missed (42%) | Patients requested or loved the concept (18%) | Detection of sessile serrated lesions / improved ADR / improved procedure time (all 6%) |  |
|  |  |  |  |  | |
| **Tham et al 2023** | Improve the quality of colonoscopy (75%) |  |  |  |  |
| **Tian et al 2022** | Improve the accuracy (sensitivity and specificity) of the diagnosis | Make the endoscopists concentrate more on the procedure | Accurately identify blind spots | Will increase the patient’s confidence in the endoscopist’s diagnosis | Make the endoscopists more interested in performing endoscopy |
| **Van der Zander et al 2022** | Improving quality of care (90%) | Time saving for the physicians (55%) | Faster diagnosis and shorter waiting times for the patients (51%) | Personalized care (27%) | Solutions for complex care tasks (21%) |
| **Wadhwa et al 2020** | Increase number of polyps removed (80.6%) | Increase ADR (76%) | Higher endoscopist satisfaction (64%) | Feel comfortable leaving hyperplastic polyps (57%) | Higher patient satisfaction (36%) |
| **Watkins et al 2024** | Easy to use (100%) | Help focus on exposing all the colonic mucosa (48%) | Provide comfort by providing a second pair of eyes (44%) | Improve overall performance (43%) | Find a clinically meaningful number of polyps missed (10%) |

AI: artificial intelligence; ADR: adenoma detection rate; PDR: polyp detection rate; EMR: electronic medical record

Ranking was determined based on the proportion (%) of respondents who voted for the corresponding advantage item.

| **Supplemental Table 7.** Top perceived disadvantages and barriers for adopting AI-assisted colonoscopy   \| **Study** \| **Rank** \| \| \| \| \| \| --- \| --- \| --- \| --- \| --- \| --- \| \| 1 \| 2 \| 3 \| 4 \| 5 \| \| **Kader et al 2022** \| Lack of guidelines (92%) \| Access to AI devices (89%) \| Availability of Devices with regulatory approval (88%) \| Accountability for incorrect diagnosis (85%) \| Evidence for cost-effectiveness (84%) \| \| **Kochar et al 2021** \| Replacement of physicians by machines (37%) \| Data/patient information security (32%) \| Become less efficient in caring for patients (18%) \| Increase workload (12%) \| Make physicians obsolete in caring for patients (8%) \| \| **Leggett et al 2024** \| Makes GI duties more technical (47%) \|  \|  \|  \|  \| \| **Nazarian et al 2023** \| Cost (64%) \| Accessibility (56%) \| Lack of guidelines (51%) \| Lack of research (35%) \| Data ownership (27%) \| \| **Nehme et al 2023**  Pre-implementation  Post-implementation \| Too many false-positive signals (69%) \| Unnecessarily longer procedure time (37%) \| Too distracting (25%) \| Not worthwhile improvement in ADR (25%) \| Medicolegal concern / Too expensive (both 12%) \| \| Too many false-positive signals (82%) \| Too distracting (59%) \| Prolonged procedure time (47%) \| Audio beep too load (41%) \| Only found obvious lesions (12%) \| \| **Tham et al 2023** \| Reduces the jobs available to providers (19%) \|  \|  \|  \|  \| \| **Tian et al 2022** \| Less responsibility for medical negligence \|  \|  \|  \|  \| \| **Van der Zander et al 2022** \| Insufficiently developed IT infrastructure (56%) \| Lack of (technical) knowledge by physicians (50%) \| Responsibility (uncertainty about laws and regulations) (35%) \| Costs (25%) \| Lack of human supervision (25%) \| \| **Wadhwa et al 2020** \| Cost (75%) \| Operator dependence (63%) \| Increased procedure time (60%) \| Higher number of false positive detections (34%) \|  \| \| **Watkins et al 2024** \| Frequent false positive detection (44%) \| Green boxes are bothersome when there is not a polyp (43%) \| Sound that goes along with the green box is bothersome (40%) \| Monitoring quality metrics may be used against the provider (29%) \| Variability in CADe ability to detect polyps (17%) \|   AI: artificial intelligence; ADR: adenoma detection rate; PDR: polyp detection rate; GI: Gastroenterology  Ranking was determined based on the proportion (%) of respondents who voted for the corresponding disadvantage item.  **Supplemental table 8. Quality appraisal of studies** | | | | | | | | | | | |
| --- | --- | --- | --- | --- | --- | --- | --- | --- | --- | --- | --- | --- | --- | --- | --- | --- | --- | --- | --- | --- | --- | --- | --- | --- | --- | --- | --- | --- | --- | --- | --- | --- | --- | --- | --- | --- | --- | --- | --- | --- | --- | --- | --- | --- | --- | --- | --- | --- | --- | --- | --- | --- | --- | --- | --- | --- | --- | --- | --- | --- | --- | --- | --- | --- | --- | --- | --- | --- | --- | --- | --- | --- | --- | --- | --- | --- | --- | --- | --- | --- | --- | --- | --- | --- | --- | --- | --- |
| Study | Joanna Briggs Institute critical appraisal checklist items* | | | | | | | | | | |
|  | Item 1 | Item 2 | Item 3 | Item 4 | Item 5 | Item 6 | Item 7 | Item 8 | Item 9 | Item 10 | Overall |
| Goh et al 2024 | Yes | Yes | Yes | Yes | Yes | n/a | Yes | Yes | Yes | Yes | Low |
| Kader et al 2022 | Yes | Yes | Yes | Yes | Yes | n/a | Yes | Yes | No | Yes | Low |
| Kochhar et al 2021 | Yes | Yes | Yes | Yes | Yes | n/a | Yes | No | Yes | Yes | Low |
| Leggett et al 2024 | Yes | Yes | Yes | Yes | Yes | n/a | Yes | Yes | Yes | Yes | Low |
| Nazarian et al 2023 | Yes | Yes | Yes | No | Yes | n/a | Yes | Yes | Yes | Yes | Low |
| Nehme et al 2023 | Yes | Yes | Yes | Yes | Yes | n/a | Yes | Yes | Yes | Yes | Low |
| Schulz et al 2023 | Yes | Yes | Yes | Yes | Yes | n/a | Yes | Yes | Yes | Yes | Low |
| Tham et al 2023 | Yes | Yes | Yes | Yes | Yes | n/a | Yes | Yes | No | Yes | Low |
| Tian et al 2022 | Yes | Yes | Yes | Yes | Yes | n/a | Yes | Yes | Yes | Yes | Low |
| Van der Zander et al 2022 | Yes | Yes | Yes | Yes | Yes | n/a | Yes | Yes | Yes | Yes | Low |
| Van der Zander et al 2024 | Yes | Yes | Yes | Yes | Yes | n/a | Yes | Yes | Yes | Yes | Low |
| Wadhwa et al 2020 | Yes | Yes | Yes | Yes | Yes | n/a | No | Yes | Yes | Yes | Low |
| Watkins et al 2024 | Yes | Yes | Yes | Yes | Yes | n/a | Yes | No | Yes | Yes | Low |

n/a: not ascertained.

*Quality appraisal was evaluated using the Joanna Briggs Institute (JBI) critical appraisal checklist for Qualitative Research (<https://jbi.global/sites/default/files/2021-10/Checklist_for_Qualitative_Research.docx>). Check list items:

1. Congruity between the stated philosophical perspective and the research methodology; 2. Congruity between the research methodology and the research question or objectives; 3. Congruity between the research methodology and the methods used to collect data; 4. Congruity between the research methodology and the representation and analysis of data; 5. There is congruence between the research methodology and the interpretation of results; 6. Locating the researcher culturally or theoretically; 7. Influence of the researcher on the research, and vice-versa, is addressed; 8. Representation of participants and their voices; 9. Ethical approval by an appropriate body; 10. Relationship of conclusions to analysis, or interpretation of the data.

**Figure 1. Literature search and study selection.** The Preferred Reporting Items for Systematic Reviews and Meta-analysis (PRISMA) flowchart for literature search and selection of the final studies included.

*Adapted from:*  Page MJ, McKenzie JE, Bossuyt PM, Boutron I, Hoffmann TC, Mulrow CD, et al. The PRISMA 2020 statement: an updated guideline for reporting systematic reviews. BMJ 2021;372: n71. doi: 10.1136/bmj. n71. For more information, visit: <http://www.prisma-statement.org/>
